# Brain MRI findings in near-term and term infants admitted to a neonatal ward

**DOI:** 10.3389/fneur.2026.1792288

**Published:** 2026-08-03

**Authors:** Xiaoling Hu, Yubing Chen, Wenwen Chen, Licong Lin

**Affiliations:** Zhangzhou Affiliated Hospital of Fujian Medical University, Zhangzhou, China

**Keywords:** asymptomatic, brain injury, MRI, newborn, non-parenchymal hemorrhage

## Abstract

**Objective:**

This study aimed to determine the incidence and patterns of abnormalities detected on brain magnetic resonance imaging (MRI) in near-term and term infants admitted to the neonatal ward.

**Methods:**

A retrospective analysis was conducted of 714 near-term or term neonates (≥36 weeks' gestation) who were admitted to a tertiary neonatal ward between January 2023 and December 2023 and who underwent brain MRI scans.

**Results:**

Abnormal MRI findings were observed in 254 of 714 (35.6%) infants. The most common abnormalities were non-parenchymal hemorrhage, present in 162 of 714 (22.7%) infants, including subarachnoid hemorrhage (20.9%), cephalohematoma (11.2%), subdural hemorrhage (5.6%), extradural hemorrhage (5.6%), and intraventricular hemorrhage (2.8%), followed by brain parenchymal injury, present in 102 of 714 (14.3%) infants. Non-cerebral parenchymal hemorrhage and brain parenchymal injury were more frequently observed after vaginal deliveries and were associated with resuscitation with positive-pressure ventilation at birth and hypoxic-ischemic encephalopathy. Subependymal cysts were observed in 31 of 714 (4.3%) infants and were not associated with perinatal insults. Congenital variations or deformities were found in 8 of 714 (1.1%) infants.

**Conclusion:**

Abnormal brain MRI findings were common in infants admitted to the neonatal ward after birth due to perinatal risk factors. The association between these MRI-defined cerebral injuries and long-term neurodevelopmental outcomes remains unclear and should be explored in long-term longitudinal studies using quantitative volumetric techniques to assess MRI-defined brain injuries and more sensitive instruments to address cognitive function.

## Introduction

Magnetic resonance imaging (MRI) is a safe and universal modality for evaluating neonatal neurological conditions. It is widely used to identify potential cerebral lesions in infants at high risk of brain injury. The majority of previous studies evaluating brain injury have focused primarily on preterm infants, particularly those born very or extremely preterm (1). Near-term and term infants comprise the majority of annual births worldwide (2). Some of these infants require postnatal monitoring in neonatal wards or medical intervention due to respiratory insufficiency, asphyxia, infection, hypoglycemia, feeding problems, or other conditions. Whether these infants are more prone to brain injury is of considerable interest to neonatologists because this may indicate an increased risk of suboptimal neurodevelopmental outcomes (3). Looney et al. found that asymptomatic intracranial hemorrhage (ICH) following vaginal birth in full-term neonates was common, with an estimated prevalence of 26% (4). However, the prevalence of other abnormalities in asymptomatic neonates and whether these abnormalities are associated with perinatal factors remain unclear. This study aimed to assess the incidence and patterns of abnormalities detected on brain MRI in near-term and term infants admitted to the neonatal ward and to examine the association between perinatal risk factors, neonatal conditions, and brain MRI findings in these infants.

## Methods

This was a retrospective study of near-term and term neonates (≥36 weeks of gestational age) who were admitted to our neonatal ward between January 2023 and December 2023 and who underwent brain MRI scans using data from the computerized medical record database. This study was approved by the Institutional Review Board (IRB) of Zhangzhou Affiliated Hospital of Fujian Medical University, with a waiver of informed consent (2024LWB281).

MRI scans were performed on a 1.5T system. The MRI scan sequence included T1-weighted imaging (T1WI), T2-weighted imaging (T2WI), diffusion-weighted imaging (DWI), and susceptibility-weighted imaging (SWI). Infants were fully fed, swaddled, and fitted with ear plugs and ear muffs throughout the MRI process. Image reports were interpreted by expert neuroradiologists. Radiologic abnormalities were classified as abnormal signal intensities on T1WI, T2WI, SWI, and DWI or structural deformities identified on T1WI and T2WI. Specifically, high signal intensity on T1WI, low signal intensity on T2WI, or low signal intensity on DWI or SWI, after excluding vascular signals, was classified as hemorrhage. Similarly, high signal intensity on T1WI, low signal intensity on T2WI, or high signal intensity on DWI was classified as non-hemorrhagic injury. Cystic lesions were classified as high signal intensity on T2WI with normal signal intensity on T1WI, DWI, and SWI.

Statistical analysis was performed using SPSS version 26.0 (IBM Corporation, USA). Non-parametric data were presented as median values with interquartile range (IQR) or range, as specified in the text, and comparisons were performed using the Mann–Whitney *U* test. The chi-square test was used to compare proportions. Statistical significance was defined as a *p*-value of < 0.05.

## Results

In 2023, our hospital had 7,918 deliveries in the obstetrics department and 3,965 admissions to the neonatology department. Common indications for admission to the neonatal department included intrauterine growth restriction (IUGR), preterm birth, perinatal asphyxia, scalp hematoma and birth injury, hypoglycemia, seizures, fever, hemorrhagic rash, suspected or confirmed sepsis, tachypnea, cyanosis, respiratory distress, pneumonia, hyperbilirubinemia, congenital malformations, and feeding problems. A total of 3,228 near-term or term infants with a gestational age (GA) of ≥36 weeks were transferred to our neonatal department during the study period. The majority of these infants (3,216 infants, 99.6%) recovered from their primary illness and were discharged home. Treatment was withdrawn in 13 infants at the request of their families due to severe illness, including congenital myopathy (two cases), congenital malformations (three cases), critical congenital heart disease (four cases), and severe encephalopathy (four cases). Indications for brain MRI included preterm birth, IUGR or small for gestational age (SGA), severe hyperbilirubinemia, perinatal asphyxia, hypoxic-ischemic encephalopathy (HIE), scalp hematoma, hypoglycemia, purulent meningitis, seizures, severe respiratory distress, and abnormalities of the nervous system structure identified on fetal ultrasound.

Ultimately, a total of 714 infants underwent MRI examinations during their hospital stay. Among them, 534 were term infants born at a GA of ≥37 weeks, and 180 were near-term infants born at a GA between 36 + 0 and 36 + 6 weeks. The age at MRI ranged from 1 day to 16 days, with a median age of 5 days. Abnormal MRI findings were identified in 254 infants, accounting for about one-third (35.6%) of all scans. Among these infants, 18 infants had apparent neurological symptoms, including seizures, altered consciousness, abnormal tone and reflexes, and poor sucking, while 236 infants had no neurological symptoms. Asymptomatic abnormal MRI findings were common, with a prevalence of 34.5% (236 abnormalities in 685 asymptomatic infants).

### Non-cerebral parenchymal hemorrhage

**Figure 1:**
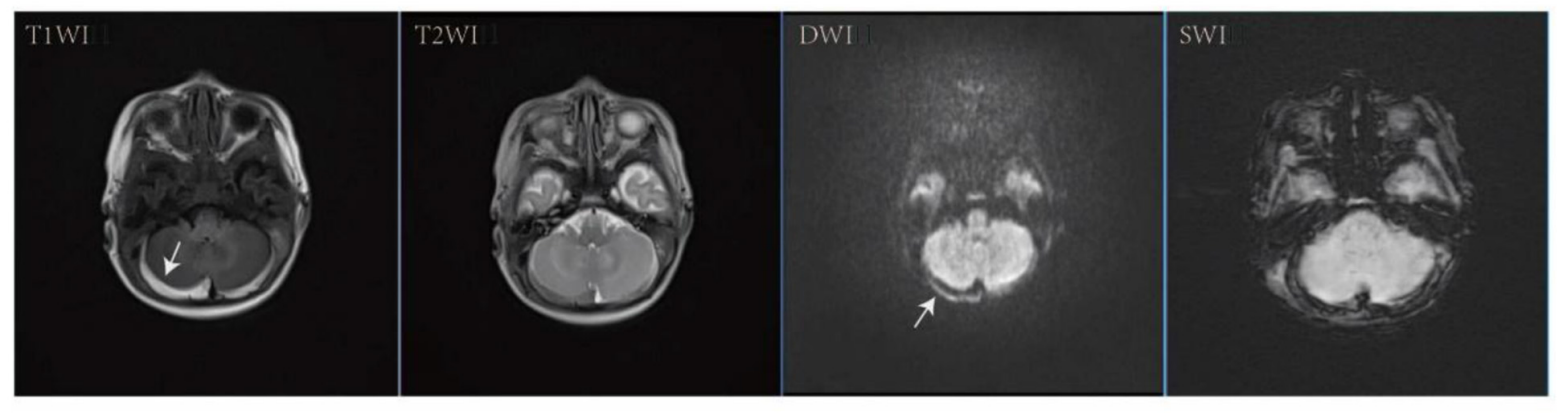
MRI images of subarachnoid hemorrhage. T1WI showed increased signal intensity in sulcus for occipital sinus. T2WI and DWI showed decreased or loss of signal intensity in the same localization.

The most common abnormalities were non-cerebral parenchymal hemorrhages, identified in 22.7% (162/714) among all scans and accounting for 63.8% (162/254) of the abnormalities. These lesions were characterized by high signals on T1WI, low signals on T2WI, or low signals on DWI or SWI (Figure 1). Among the cases of non-cerebral parenchymal hemorrhage, subarachnoid hemorrhage was the most common type (20.9%, 149/714), followed by cephalohematoma (11.2%, 8/714), subdural hemorrhage (5.6%, 4/714), extradural hemorrhage (5.6%, 4/714), and intraventricular hemorrhage (2.8%, 2/714). Maternal demographics and newborn clinical characteristics were assessed in the non-cerebral parenchymal hemorrhage and non-hemorrhage groups. The mean GA and birth weight were higher in the non-cerebral parenchymal hemorrhage group than in the non-hemorrhage group. The non-cerebral parenchymal hemorrhage group had a lower rate of fetal distress *in utero* (9.3% and 15.6%, respectively, *P* = 0.042) and a higher rate of vaginal delivery (65.8% and 29.2%, respectively, *P* < 0.001), a need for positive pressure ventilation during resuscitation at birth (13.0% and 7.6, *p* = 0.035), presence of neurological symptoms (7.4% and 3.1%, *p* = 0.014), and diagnosis of HIE (6.8% and 4.2%, *p* = 0.006). There was no significant difference between the two groups in terms of neonatal severe respiratory disease (Table 1).

**Table 1:** Maternal demographics and newborn clinical characteristics between the non-cerebral parenchymal hemorrhage and the non-hemorrhage groups.

Factors	Non-cerebral parenchymal hemorrhage group (*n* = 162)	Non-hemorrhage group (*n* = 552)	*P*-value
GA (mean ± SD, weeks)	38.5 ± 1.4	38.1 ± 1.4	0.005
BW (mean ± SD, g)	3,091 ± 589	2,895 ± 587	< 0.001
SGA *n* (%)	28 (17.3)	127 (23.0)	0.120
Male *n* (%)	91 (56.2)	291 (52.1)	0.438
Gestational hypertension *n* (%)	8 (4.9)	47 (8.5)	0.133
GDM *n* (%)	46 (28.4)	162 (29.3)	0.814
Hypothyroidism in pregnancy *n* (%)	16 (9.9)	83 (15.0)	0.095
Fetal distress *n* (%)	15 (9.3)	86 (15.6)	0.042
Vaginal delivery *n* (%)	106 (65.8)	161 (29.2)	< 0.001
Resuscitation with positive pressure ventilation at birth *n* (%)	21 (13.0)	42 (7.6)	0.035
Neurological symptoms *n* (%)	12 (7.4)	17 (3.1)	0.014
HIE *n* (%)	11 (6.8)	13 (2.4)	0.006
Respiratory distress *n* (%)	20 (12.3)	63 (11.4)	0.745

GA, gestational age; BW, birth weight; SGA, small for gestational age; GDM, gestational diabetes mellitus; HIE, hypoxic-ischemic encephalopathy.

HIE was diagnosed according to the Sarnat stages (33).

### Brain parenchymal injury

**Figure 2:**
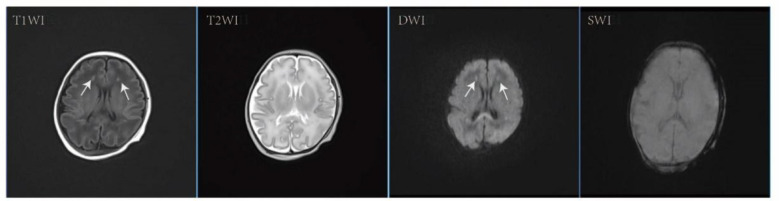
MRI images of punctate white matter injuries. T1WI and DWI showed increased signal intensity near the anterior horn of the lateral ventricle. T2WI showed decreased or loss of signal intensity in the same localization.

Neonatal brain parenchymal injury typically consists of hemorrhagic and ischemic lesions, which can be identified as abnormal signal intensities in the parenchyma, appearing as T1 high signals, T2 low signals, or high signals on DWI (5) (Figure 2). A total of 102 infants had brain parenchymal injury confirmed by MRI, with a prevalence of 14.3% (102/714) among all MRI examinations and 40.2% (102/254) among those with abnormal findings. The most common injury pattern was white matter injury (WMI), with a prevalence of 69.6% (71/102), followed by watershed-pattern injury (12.7%, 13/102), cerebellar hemorrhage (6.9%, 7/102), the basal ganglia-thalamus (BGT) injury pattern (3.9%, 4/102), corpus callosum injury (2.9%, 3/102), and perinatal arterial ischemic stroke (1.0%, 1/102). Six infants (5.9%, 6/102) had high signal in the globus pallidus and subthalamic nucleus on T1WI as well as a subtle increased signal on T2WI, which suggested the possibility of bilirubin-induced brain injury.

There were no differences in GA, BW, and sex between the brain parenchymal injury group and the non-injury group. Maternal hypertension, GDM, and hypothyroidism were not associated with neonatal brain parenchymal injury. Infants in the brain parenchymal injury group had a lower rate of vaginal delivery (46.5% and 65.1%, respectively, *P* = 0.014), a higher rate of receiving positive pressure ventilation during resuscitation at birth (15.9% and 7.8%, respectively, *P* < 0.001), a lower rate of being SGA (14.1% and 23.0%, respectively, *P* = 0.035), a higher rate of neurological symptoms (8.8% and 3.3%, respectively, *P* = 0.018), and a higher rate of HIE diagnosis (8.8% and 2.5%, respectively, *P* = 0.003; Table 2). No significant difference was detected between the two groups in terms of neonatal severe respiratory disease (Table 2).

**Table 2:** Maternal demographics and newborn clinical characteristics of the brain parenchymal injury and the non-injury groups.

Factors	Brain parenchymal injury (*n* = 102)	Non-injury (*n* = 612)	*P*
GA (mean ± SD, weeks)	38.2 ± 1.5	38.2 ± 1.4	0.713
BW (mean ± SD, g)	3,004 ± 520	2,929 ± 604	0.236
SGA *n* (%)	14 (14.1)	137 (23.0)	0.035
Male *n* (%)	58 (56.9)	324 (52.9)	0.462
Gestational hypertension *n* (%)	3 (2.9)	52 (8.5)	0.051
GDM *n* (%)	27 (26.5)	181 (29.6)	0.523
Hypothyroidism in pregnancy *n* (%)	13 (12.7)	876 (14.1)	0.724
Fetal distress in the uterus *n* (%)	13 (12.7)	88 (14.4)	0.661
Vaginal delivery *n* (%)	47 (46.5)	398 (65.1)	< 0.001
Resuscitation with positive pressure ventilation at birth *n* (%)	16 (15.7)	47 (7.7)	0.008
Neurological symptoms *n* (%)	9 (8.8)	20 (3.3)	0.018
HIE *n* (%)	9 (8.8)	15 (2.5)	0.003
Respiratory distress *n* (%)	16 (15.7)	67 (10.9)	0.167

GA, gestational age; BW, birth weight; SGA, small for gestational age; GDM, gestational diabetes mellitus; HIE, hypoxic-ischemic encephalopathy.

HIE was diagnosed according to the Sarnat stages (33).

### Subependymal cyst

**Figure 3:**
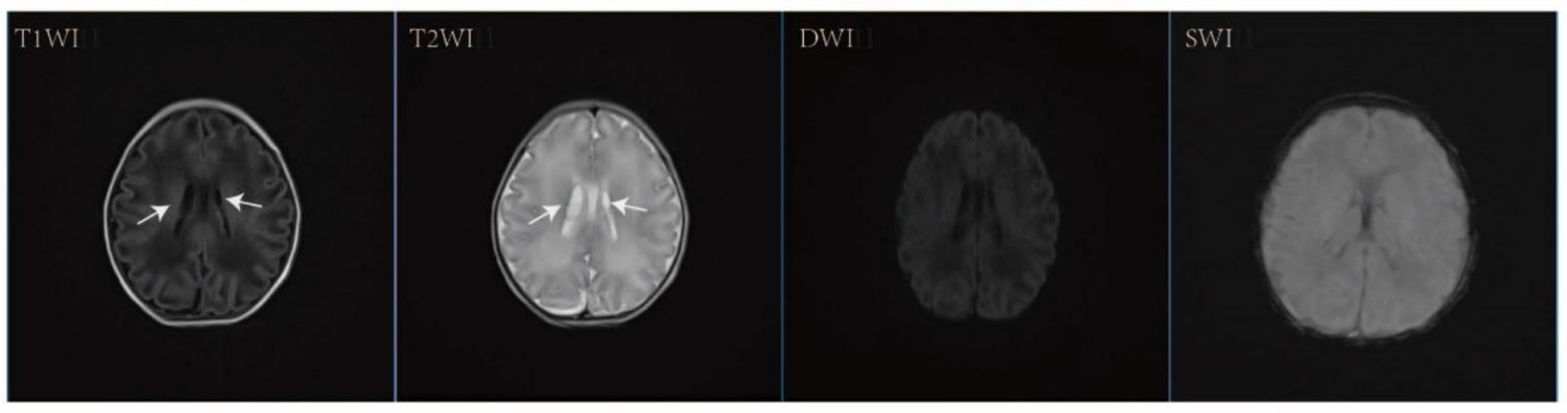
MRI images of subependymal cyst located in the body of the lateral ventricle.

Thirty-one infants were found to have subependymal cysts visualized by MRI, with a prevalence of 4.3% (31/714) among all MRI scans and 12.2% (31/254) among those with abnormal findings, appearing as cystic lesions in the subependymal germinal matrix (Figure 3). More than half of the subependymal cysts (67.7%, 21/31) were bilateral, and the remaining unilateral subependymal cysts were all located on the left side (100%, 10/10). There were no differences in maternal demographics and newborn clinical characteristics between the subependymal cyst and the non-cyst groups (Table 3).

**Table 3:** Maternal demographics and newborn clinical characteristics of the subependymal cyst and the non-cyst groups.

Factors	Subependymal cyst (*n* = 31)	Non-cyst (*n* = 683)	*P*
GA (mean ± SD, weeks)	38.1 ± 1.6	38.2 ± 1.4	0.831
BW (mean ± SD, g)	3,100 ± 582	2,935 ± 594	0.338
SGA *n* (%)	4 (12.9)	151 (22.1)	0.224
Male *n* (%)	15 (48.4)	367 (53.7)	0.559
Gestational hypertension *n* (%)	3 (9.7)	52 (7.6)	0.938
GDM *n* (%)	6 (19.4)	202 (29.6)	0.221
Hypothyroidism in pregnancy *n* (%)	3 (9.7)	96 (14.1)	0.671
Fetal distress in the uterus *n* (%)	8 (25.8)	93 (13.6)	0.101
Vaginal delivery *n* (%)	16 (51.6)	251 (36.9)	0.097
Resuscitation with positive pressure ventilation at birth *n* (%)	1 (3.2)	62 (9.1)	0.424
Neurological symptoms *n* (%)	0 (0)	29 (4.2)	0.480
HIE *n* (%)	0 (0)	24 (3.5)	0.581
Respiratory distress *n* (%)	3 (9.7)	80 (11.7)	0.953

GA, gestational age; BW, birth weight; SGA, small for gestational age; GDM, gestational diabetes mellitus; HIE, hypoxic-ischemic encephalopathy.

HIE was diagnosed according to the Sarnat stages (33).

### Congenital variation or deformity

**Figure 4:**
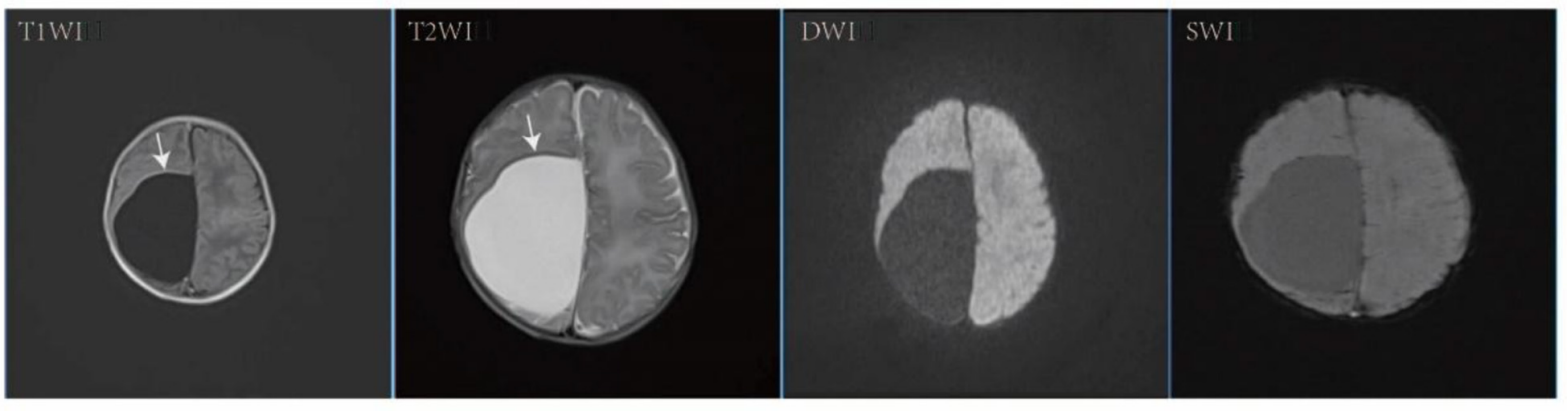
MRI images of a large parieto-occipital cyst.

Arachnoid cysts (Figure 4) were present in six infants (0.8%, 6/714). One infant was found to have agenesis of the corpus callosum. One infant was found to have a vascular malformation in the right occipital lobe. The overall rate of congenital variations or deformities was 1.1% (8/714).

Follow-up information was extracted from the outpatient electronic medical record system for 591 cases. At the time of publication, 33 cases had apparent neurodevelopmental disorders. There was no significant difference between the MRI abnormal group and the normal group in terms of neurological disorders, either overall or among asymptomatic infants.

## Discussion

Owing to its non-invasiveness, lack of radiation, clear contrast, and high spatial resolution, MRI is more sensitive and specific for detecting brain abnormalities, which is favored by neonatologists for the evaluation of the developmental brain. As shown in this survey, MRI was frequently performed in our neonatal ward among near-term and term infants despite the absence of obvious symptoms. A routine MRI scan includes T1, T2, SWI, and DWI sequences. T1- and T2-weighted sequences are the basis for determining anatomy and morphological alterations. Generally, T1WI sequences have high sensitivity for hemorrhage and lipid content, while T2WI sequences have high sensitivity for water (tissue edema) (6). SWI sequences are useful for detecting bleeding. DWI can help to detect cytotoxic edema (7). Numerous studies have demonstrated that MRI findings were meaningful for predicting neurodevelopmental outcomes and making clinical decisions. However, incidental findings were common in asymptomatic neonates. The prevalence of abnormal findings in hospitalized asymptomatic near-term and term infants was 34.5% in our study, while Carney O et al. found a slightly higher prevalence of 47% (8). It is worth noting that infants admitted to the neonatal ward almost always had abnormal perinatal history or laboratory test indicators (IUGR, respiratory problems, suspicious hypoxia or infection, difficult labor, hypoglycemia, hyperbilirubinemia, etc.) that may be related to central nervous system (CNS) injury. Thus, the incidence of brain MR abnormalities, including hemorrhage or parenchymal injury, in the study population is not the same as that in the general population. The percentages would likely be much lower in a general birth population. The interpretation of these abnormalities should be cautious.

In our study, non-cerebral parenchymal hemorrhage was the most common abnormal finding, accounting for more than half (63.8%, 162/254) of the overall abnormalities, with a prevalence of 22.7% among all scans. Previous studies have also found a wide distribution of intracranial hemorrhage in term and late-preterm infants (9), among which subarachnoid hemorrhage and subdural hemorrhage were the most common types (10). In our study population, subarachnoid hemorrhage was the most common type of non-cerebral parenchymal hemorrhage. However, Krishnan et al. found that subdural hemorrhage was the most common type, occurring in more than one-third of normal deliveries (11). This difference may be due to the fact that we completed the MR imaging at an earlier age (ranging from 1 to 16 days, with a median age of 5 days), while Krishnan et al. conducted MRI with an age range of 11–25 days. The cause of non-cerebral parenchymal hemorrhage is non-specific, and it is probably complicated by birth trauma, asphyxia, acidosis, and hypoglycemia. As revealed by our study, non-cerebral parenchymal hemorrhage was more frequently observed in infants with older GA, larger BW, and vaginal delivery. The incidence of asymptomatic hemorrhage after vaginal delivery was 38.9% (100/257), which seemed a little higher than that reported in the study of Looney et al. (26%) (4). Moreover, we also found that non-cerebral parenchymal hemorrhage might be associated with resuscitation with positive pressure ventilation at birth and subsequent HIE. A majority of the cases of non-cerebral parenchymal hemorrhage did not have obvious clinical manifestations and could spontaneously resolve with little clinical consequence (12). Only a few of the infants exhibited seizures in the presence of massive hemorrhage combined with intraventricular and parenchymal hemorrhage (13). Asymptomatic neonates with non-cerebral parenchymal hemorrhage would likely have favorable neurodevelopmental outcomes (14).

HIE remained an independent risk factor for neonatal brain injury and delayed neurodevelopment. The study revealed that infants without HIE symptoms also had a probability of brain injury. Except for the standard injury patterns in the basal ganglia, watershed, and corpus callosum in HIE, the most common injury pattern was punctate white matter injuries (PWMLs) in near-term and term infants. PWMLs can be identified on routine brain MRI scans, presenting as hyperintensity on T1WI and hypointensity on T2WI. Previous studies have found a relationship between white matter injury of the frontal lobe and cognitive outcome (15), while PWMLs were associated with motor outcome (16). Hayman M et al. found that PWMLs in the setting of a genetic disorder were associated with the highest risk of a poor outcome (17). These findings warranted consultation and close follow-up with neurology.

The prevalence of subependymal cysts was 4.3% (31/714) in near-term and term infants, as revealed by our study, and the findings appeared unrelated to the common perinatal risk factors. Shen EY and Huang FY reported a similar incidence of 5% (18). These brain lesions have also been observed antenatally (19). Subependymal cysts were usually described as “pseudocysts” indicative of concealed intrauterine subependymal hemorrhage or germinolysis lined with immature, undifferentiated cells, which were most commonly located in the germinal matrix of the subependymal lining of the lateral ventricles (20). The most common forms were frontal horn cysts. It is important to distinguish between pseudocysts and cystic periventricular leukomalacia, as cystic periventricular leukomalacia invariably leads to cerebral palsy and/or visual impairment (21), while pseudocysts are presented as a benign pathologic condition that could be expected to resolve spontaneously within a few months, regardless of the location, number, or size (22). For pseudocysts with additional findings, the neurodevelopmental outcomes might not be optimistic. The visualization of subependymal cysts should lead to a detailed evaluation, including maternal TORCH status, additional brain findings, malformations of other organs, karyotyping/comparative genomic hybridization, and genetic counseling to make a precise interpretation (23).

Arachnoid cysts are one of the most common congenital abnormalities in fetuses and newborns, which might be found via prenatal ultrasound or MRI screenings. Arachnoid cysts could regress spontaneously or remain stable (24). The conventional management of arachnoid cysts was performing endoscopic fenestration in the first week of life. Final surgical therapy consisted of cyst marsupialization and/or cysto-peritoneal shunt implantation (25). In general, the neurodevelopmental outcome of arachnoid cysts is favorable (24). Yahal et al. reported that more than two-thirds (77.3%) of arachnoid cysts had normal neurodevelopmental outcomes, while less than one-third (22.7%) had impaired neurodevelopment (26).

MRI has an excellent sensitivity for detecting abnormalities in the brain. However, these abnormalities in early life appear not to have enough power to accurately predict the neurodevelopmental outcome of near-term or term neonates. Similarly, Guillot et al. pointed out that MRI had a high negative predictive value but relatively low positive predictive value in predicting neurodevelopmental outcomes (27). The neonatal brain is not only vulnerable to insults but also possesses an incredible ability to repair during its maturational process. The signal abnormalities may exhibit evolution at different time points of imaging. Thus, abnormalities at a single time point are not sufficient to predict prognosis since they may be transient. It is also important to note that there are reports of infants with neurodevelopmental disorders who do not show any abnormalities on MRI at all. When interpreting signal abnormalities on MRI, greater caution is needed. White matter injury (WMI) and deep gray matter (DGM) injury were the main patterns of brain injury observed on early MRI, which were mostly attributed to perinatal asphyxia as demonstrated by signs of fetal distress, a birth history with low Apgar scores, intensive resuscitation, or umbilical cord blood pH ≤ 7.0 (28). The implications of injury to the DGM vs. the WM on the long-term neurodevelopmental outcomes differ in the context of hypoxic-ischemic encephalopathy (29). It has been reported that DGM abnormalities owing to acute asphyxia events were associated with an increased likelihood of adverse neurodevelopmental outcomes (30). However, a growing number of studies have suggested a shift to milder forms of chronic injury in the last decade (31). The prognosis of these MRI-defined cerebral injuries remains largely undefined, since they may display more subtle disability with a broad spectrum of cognitive dysfunction that involves various aspects of learning, memory, language, vision, hearing, attention, and socialization as age increases, rather than motor disorders or cerebral palsy. Moreover, it is increasingly acknowledged that many genetic/epigenetic and nutritional factors might influence primary injury progression with potentially reversible or irreversible consequences (32). To address these issues, more precise sub-scores to grade the severity and extent of WM or DGM injury, more sensitive instruments to identify cognitive function in the asymptomatic population, and dynamic assessment techniques should be considered.

The main strengths of our study included the large sample size of 714 near-term and term neonates based on conventional MR imaging. The current data provide an important insight into brain MRI abnormalities in the asymptomatic neonatal population. The main limitation was the lack of quantification in the conversion of MRI images into mineable data. In addition, a challenge of our retrospective study was the collection of adequate information to validate the neurodevelopmental outcomes.

## Conclusion

Abnormal brain MRI findings were common in infants who were admitted to the neonatal ward after birth due to perinatal risk factors. The association between these MRI-defined cerebral injuries and long-term neurodevelopmental outcomes remains unclear and should be explored with long-term longitudinal studies using quantitative volumetric techniques to grade MRI brain injuries and more sensitive instruments to address cognitive function.

## Data Availability

The raw data supporting the conclusions of this article will be made available by the authors, without undue reservation.
